# Green tea extract catechin improves internal cardiac muscle relaxation in RCM mice

**DOI:** 10.1186/s12929-016-0264-1

**Published:** 2016-06-28

**Authors:** Xiaoqin Wang, Zhengyu Zhang, Gang Wu, Changlong Nan, Wen Shen, Yimin Hua, Xupei Huang

**Affiliations:** Department of Pediatric Cardiology, West China Second University Hospital, Sichuan University, Chengdu, Sichuan 610041 China; Department of Biomedical Science, Charles E. Schmidt College of Medicine, Florida Atlantic University, 777 Glades Road, Boca Raton, FL 33431 USA

**Keywords:** Diastolic dysfunction, Restrictive cardiomyopathy, Calcium desensitization, Green tea extracts, Pressure-volume relationship, Experimental animals

## Abstract

**Background:**

Diastolic dysfunction refers to an impaired relaxation and an abnormality in a heart’s filling during diastole while left ventricular systolic function is preserved. Diastolic dysfunction is commonly observed in patients with primary hypertension, diabetes and cardiomyopathies such as hypertrophic cardiomyopathy or restrictive cardiomyopathy. We have generated a restrictive cardiomyopathy (RCM) mouse model with troponin mutations in the heart to mimic the human RCM patients carrying the same mutations.

**Results:**

In the present study, we have investigated the ventricular muscle internal dynamics and pressure developed during systole and diastole by inserting a micro-catheter into the left ventricle of the RCM mice with or without treatment of desensitizer green tea extracts catechins. Our results demonstrate that green tea catechin is able to correct diastolic dysfunction in RCM mainly by improving ventricular compliance and reducing the internal muscle rigidity caused by myofibril hypersensitivity to Ca^2+^.

**Conclusion:**

Green tea extract catechin is effective in correcting diastolic dysfunction and improving ventricular muscle intrinsic compliance in RCM caused by troponin mutations.

## Background

Diastolic dysfunction refers to an impaired relaxation and an abnormality in a heart’s filling during diastole while left ventricular systolic function is preserved. Diastolic dysfunction is a common sign in elderly population and in patients suffering from primary hypertension and various cardiomyopathies [1, 2]. For example, both hypertrophic cardiomyopathy (HCM) and restrictive cardiomyopathy (RCM) share a common feature of diastolic dysfunction [3, 4]. Diastolic dysfunction is an important clinical syndrome since almost half of heart failure patients have diastolic dysfunction [5–7]. There is a critical need to understand the mechanisms that cause diastolic dysfunction and heart failure and to develop target-based intervention and medications to correct the overt condition and to treat the patients. For the moment, in the absence of such agents and intervention, treatment of diastolic dysfunction is difficult and often ineffective [8].

In our previous studies, we have demonstrated that hypersensitivity of myofibrils to Ca^2+^ is a key factor associated with impaired relaxation in the heart [9, 10]. Furthermore, we have demonstrated, for the first time that desensitization of Ca^2+^ hypersensitivity in RCM can correct phenotypes and rescue the RCM mice [11, 12]. In applying green tea extracts catechins into RCM mice, we have further indicated that Ca^2+^ desensitizer green tea catechins can reverse diastolic dysfunction in RCM mice with troponin mutations [13]. However, it is not clear whether catechins correct the impaired relaxation by altering calcium dynamics or by altering the myofibril protein interactions, i.e. internal cardiac muscle dynamics. In the present study, we have determined the internal ventricular muscle dynamics using pressure-volume measurements in left ventricles in RCM mice with or without treatment of green tea catechins. Furthermore, the calcium handling proteins have been analyzed to exclude their effects on Ca^2+^ dynamics in RCM myocardial cells. Our results demonstrate that green tea catechin is able to correct diastolic dysfunction in RCM mainly by improving ventricular compliance and reducing the internal muscle rigidity caused by myofibril hypersensitivity to Ca^2+^.

## Methods

### Animals

RCM cTnI^193His^ transgenic mice (TG) used in this study were generated in our laboratory by transgenic expression of cTnI^193His^ mutant protein in the heart under the α-myosin heavy chain promoter [9]. Wild-type (WT) C57BL/6 mice were used as controls in the study. Both TG and WT mice were maintained in animal facilities at Florida Atlantic University. This investigation was performed in accordance with NIH guidelines, the Guide for the Care and Use of Laboratory Animals (NIH Pub. N01-OD-4-2139, 8^th^ edition, 2011) and approved by the Institutional Animal Care and Use Committees at Florida Atlantic University.

### Treatment of experimental animals

Green tea extract (-)-epigallocatechin-3-gallate (EGCg) was obtained from Sigma-Aldrich (Catalog number E4268). The compound was dissolved freshly in 15 % DMSO (200 mg/ml) as described previously [13]. Both WT and TG mice (2 months old) were injected intraperitoneally daily with EGCg solution (50 mg.kg) (5 days per week) for 3 months. Each group contained eight mice. The control mice were injected with the same amount of 15 % DMSO in a similar manner as described previously [13].

### Cardiac function measurement with high resolution echocardiography

Cardiac function in experimental mice was examined and analyzed using a Vevo 770 High-Resolution In Vivo Imaging System (VisualSonics, Toronto, ON, Canada) in our laboratory as described previously [9, 14]. To decrease experimental bias, all of the echocardiography measurements were performed by an examiner blinded to the genotype. Short-axis images were taken to view the left ventricle (LV) or right ventricle (RV) movement during diastole and systole. Transmitral blood flow was observed with Pulse Doppler. All data and images were saved and analyzed with an Advanced Cardiovascular Package Software (VisualSonics, Toronto, ON, Canada) as described previously [9, 14].

### Left ventricular pressure–volume analysis

Mice were anesthetized with 3 % isoflurane and were supported by a ventilator with a maintenance dose of 1 % isoflurane after tracheostomy. A research pressure-volume micro-catheter (1F PV catheter, Millar, Inc. Houston, TX) was inserted into the left ventricle through the right carotid artery of the experimental mice. Intra-cardiac pressure (P) and volume (V) were simultaneously measured and real time data were displayed and recorded using a P–V conductance system (MPVS Ultra, AD Instruments, Inc. Houston, TX) coupled to a digital converter (PowerLab, AD Instruments). Heart rate was continuously monitored using either ECG electrodes or direct pressure pulses corresponding to each heartbeat. Hemodynamic parameters were measured under different preloads, which were elicited by transiently compressing the abdominal inferior vena cava. The catheter was advanced into the LV to obtain +d*P*/d*t*max as a measure of systolic function. For diastolic function, LV end-diastolic pressure (LVEDP), −d*P*/d*t*min, and Tau were determined. Tau was determined from fitting to the pressure decay curve from the time when −d*P*/d*t*min occurs using a zero asymptote. At least eight mice were measured for each group. In β-adrenergic stimulation assay, bolus injection of the β-receptor agonist isopretelenol (1.5 mg/kg body wt). Assays were performed on five TG and five WT mice.

### Western blotting

Cardiac samples were resolved in NuPAGE 4–12 % gradient BT gels using an Xcell II sure lock Mini Cell gel system from Invitrogen. The proteins were transferred to a nitrocellulose membrane using the Xcell IITM blot module as described previously [9, 10]. The nitrocellulose membrane was blocked with 5 % dry fat milk in TBS-T and incubated with antibodies. The target proteins on immunoblots were visualized by enhanced chemiluminescence (ECL detection kit from GE Healthcare). The following primary antibodies were used: cardiac cTnI 4H6 antibody (1:20000) for cTnI protein. Rb pAb (phospho s16, 1:20000) was used to determine the phosphorylated phospholamban. Ms mAb to SERCA-2 ATPase antibody (1:600) was used to determine the SERCA-2 proteins. Ms mAb to Phosplamban antibody (1:20000) was used to determine phospholamban proteins. The protein bands were scanned by densitometry and quantified among the samples on the same blot.

### Statistical analysis

The results are presented as means ± SE. ANOVA followed by post hoc Newman–Keuls (SNK) tests and Student’s *t* test were used to determine the statistical significance. Statistical significance was set as *P* <0.05.

## Results

### Diastolic dysfunction is reversed by EGCg treatment in RCM mice

**Fig. 1:**
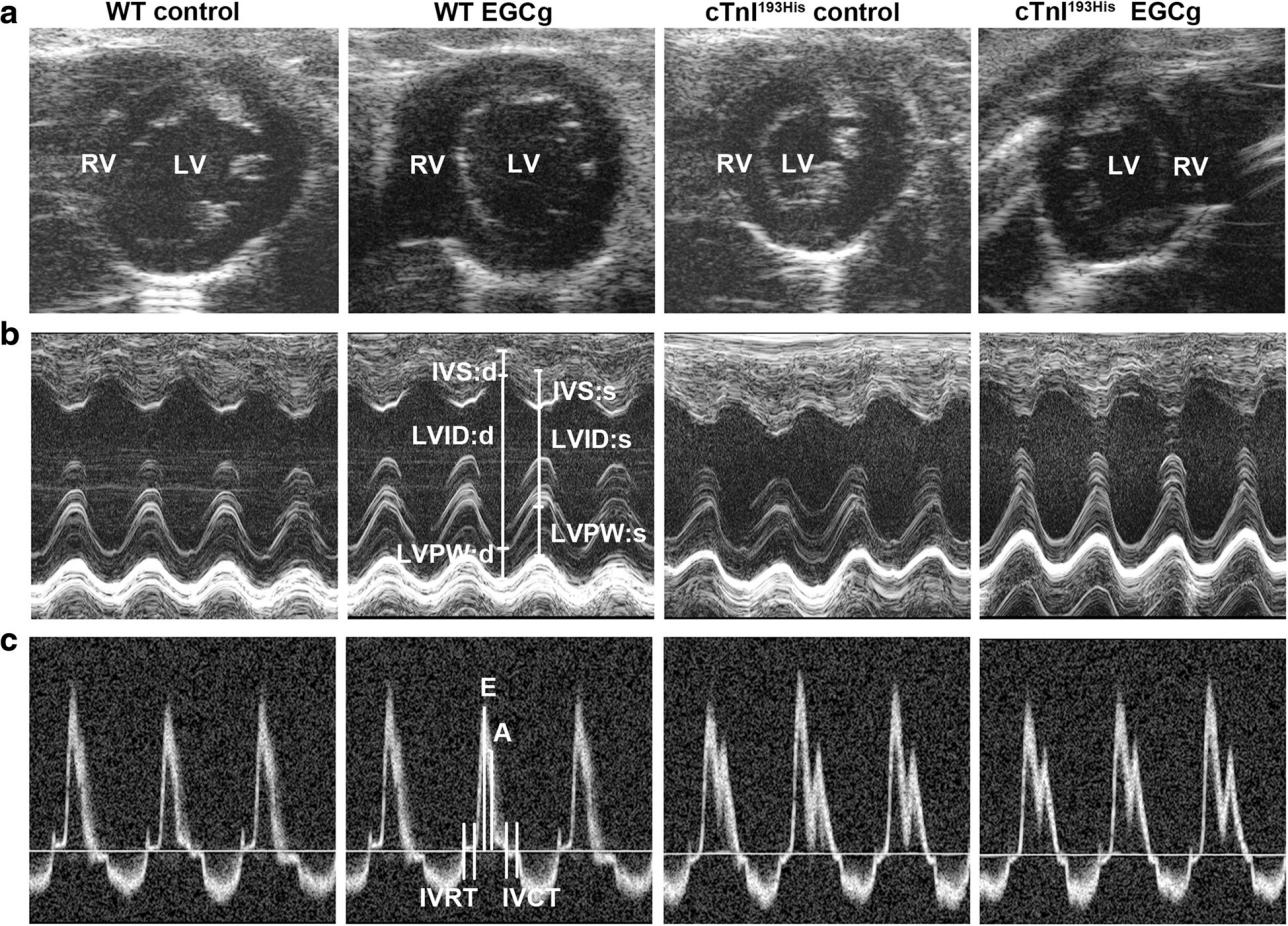
Determination of cardiac function with high resolution echocardiography in WT and RCM TG mice with or without treatment of EGCg. **a** Representative two-dimensional short axis views obtained from four different groups of the experimental mice. **b** Representative M-mode images and parameter calculation in experimental mice. **c** Representative images of pulsed Doppler of mitral inflow obtained from the experimental mice. *LV* left ventricle, *RV* right ventricle, *E* peak velocity of mitral blood inflow in early diastole, *A* peak velocity of mitral blood inflow in late diastole; E/A ratio; *IVRT* isovolumic relaxation time; *IVCT* isovolumetric contraction time, *LVID:s* left ventricular internal diameter end systole, *LVID:d* left ventricular internal diameter end diastole

The data from cardiac function measurements using high resolution echocardiography in experimental mice indicate that enlarged left and right atria are significantly reduced in RCM mice after the treatment of EGCg (Fig. 1 and Table 1). Left ventricular end diastolic dimension (LVEDD) is increased in RCM mice after the treatment. The prolonged left ventricular isovolumentric relaxation time (IVRT) is significantly shortened in RCM mice after the treatment with EGCg (Table 1). These results are consistent with our report in the previous study indicating that green tea extract EGCg is able to reverse diastolic dysfunction and improve cardiac relaxation in RCM mice.

**Table 1:** Cardiac function measurements on WT and RCM TG mice

	WT control	WT EGCg	RCM control	RCM EGCg
Parameters				
Body weight (g)	31.67 ± 1.53	30.67 ± 1.61	27.25 ± 1.21*	28.35 ± 1.28*
Heart rate (bpm)	470.72 ± 38.12	481.37 ± 44.15	450.75 ± 50.76	460.17 ± 49.73
Atria				
LAEDD (mm)	2.12 ± 0.02	2.13 ± 0.03	2.29 ± 0.04**	2.21 ± 0.05*‡
LAESD (mm)	1.67 ± 0.03	1.69 ± 0.04	1.74 ± 0.06	1.71 ± 0.05
RAEDD (mm)	2.11 ± 0.05	2.12 ± 0.06	2.31 ± 0.11**	2.26 ± 0.05*
RAESD (mm)	1.71 ± 0.06	1.69 ± 0.03	1.64 ± 0.04	1.68 ± 0.05
LV end diastole				
IVS (mm)	0.75 ± 0.04	0.76 ± 0.03	0.75 ± 0.04	0.74 ± 0.03
LVEDD (mm)	3.88 ± 0.07	3.85 ± 0.04	3.42 ± 0.0 6*	3.62 ± 0.03*‡
LV PW (mm)	0.76 ± 0.02	0.75 ± 0.04	0.76 ± 0.03	0.73 ± 0.03
LV Volume (μl)	64.67 ± 1.71	63.95 ± 2.16	46.16 ± 2.24**	55.92 ± 1.19*‡
LV end systole				
IVS (mm)	1.27 ± 0.03	1.27 ± 0.04	1.24 ± 0.02	1.25 ± 0.03
LVESD (mm)	2.05 ± 0.05	2.08 ± 0.06	1.58 ± 0.02**	1.75 ± 0.03*‡
LV PW (mm)	1.22 ± 0.03	1.22 ± 0.04	1.21 ± 0.03	1.21 ± 0.02
LV Volume (μl)	13.03 ± 0.43	13.42 ± 0.61	5.96 ± 0.71	9.35 ± 0.80**ǂǂ
Ejection Fraction (%)	79.85 ± 1.41	79.01 ± 1.00	80.33 ± 1.91	80.57 ± 2.16
Fractional Shortening (%)	46.72 ± 1.22	45.38 ± 0.66	47.95 ± 1.84	47.44 ± 2.33
Mitral Doppler				
E (mm/s)	827.61 ± 22.05	829.75 ± 24.22	740.81 ± 18.93*	809.04 ± 25.14
A (mm/s)	657.95 ± 12.34	667.35 ± 16.48	560.33 ± 33.66 *	633.57 ± 26.83
E/A	1.26 ± 0.04	1.24 ± 0.02	1.32 ± 0.05	1.27 ± 0.05
IVRT (ms)	16.41 ± 0.44	16.44 ± 0.46	21.43 ± 0.54**	18.53 ± 0.51*‡
IVCT (ms)	10.58 ± 0.33	11.05 ± 0.29	10.36 ± 0.75	10.86 ± 0.53

Values are expressed as means ± SE for each group. *LA* left atrium, *RA* right atrium, *LV* left ventricle, *EDD* end diastolic dimension, *ESD* end systolic dimension, *PW* posterior wall thickness of LV, *IVS* intra-ventricular septum, *EF* ejection fraction of LV, *FS* fractional shortening of LV, *E* mitral Doppler E peak velocity, *A* mitral Doppler A peak velocity, *IVRT* isovolumetric relaxation time, *IVCT* isovolumetric contraction time. Statistical significance was determined by ANOVA followed by post hoc Newman-Keuls (SNK) tests

*p <0.05 compared to WT control, **p <0.01 compared to WT control; ǂ*p* <0.05 compared to RCM control; ǂǂ*p* <0.01 compared to RCM control

### EGCg corrects diastolic dysfunction by reducing cardiac muscle internal rigidity and increasing ventricular compliance in RCM mice

**Fig. 2:**
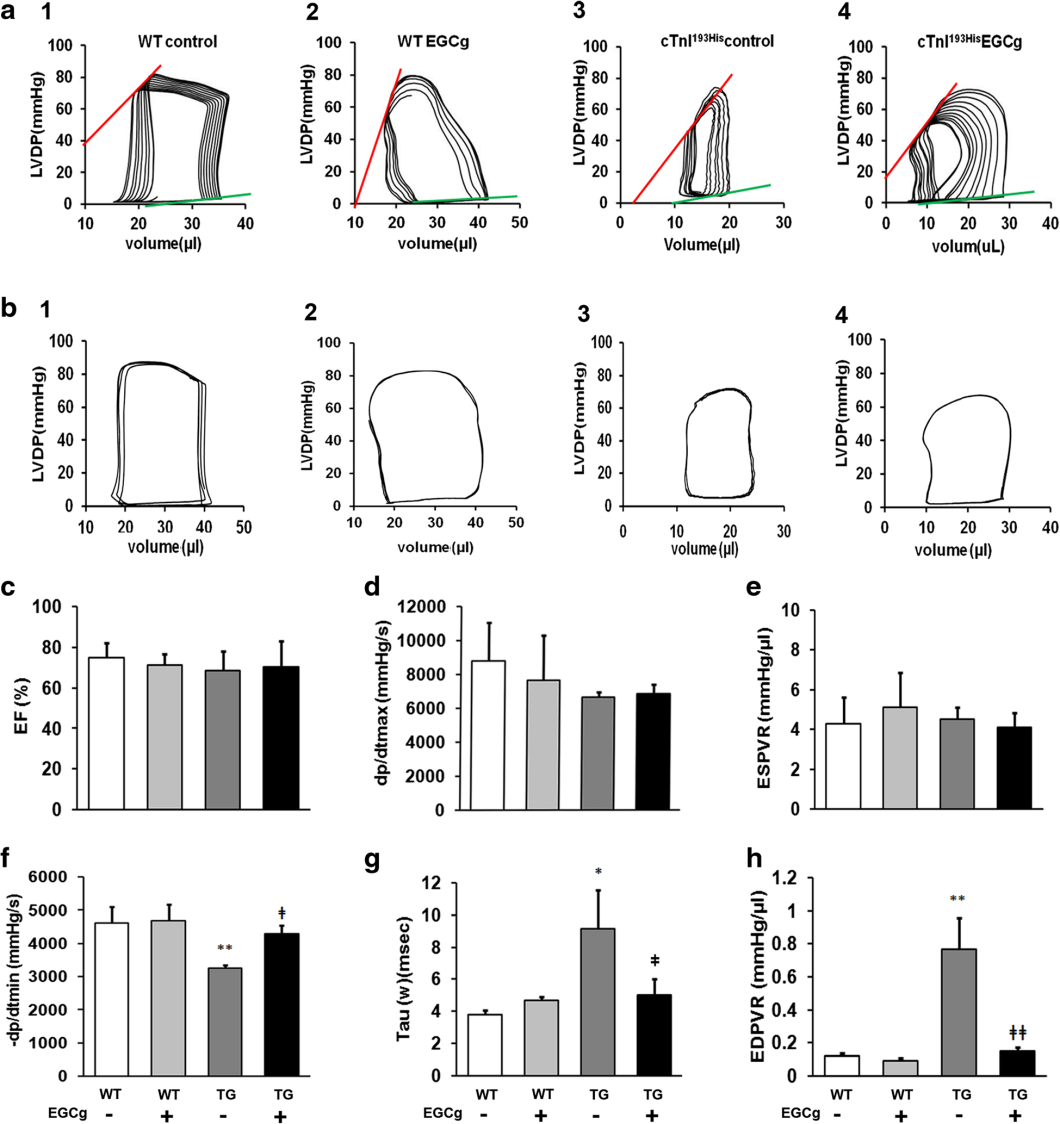
Representative pressure-volume loops obtained from catheter-based left ventricular P-V measurements in experimental mice. **a** Characteristic changes in left ventricular developmental pressure (LVDP) corresponding to the volume changes. Upper line indicates end systole pressure volume relationship (ESPVR) and the low line indicates end diastole pressure volume relationship (EDPVR) in different groups of mice: 1, WT mice in control group (WT control); 2, WT mice with EGCg treatment (WT EGCg); 3, cTnI^193His^ RCM mice in control (cTnI^193His^ control)^;^ 4, cTnI^193His^ RCM with EGCg treatment (cTnI^193His^ EGCg). **b** Normal baseline PV loops from different groups of mice. Cardiac function parameters are shown in (**c**), ejection fraction (EF); (**d**) the maximal rate of contraction (+d*P*/d*t*); (**e**) end-systolic pressure–volume relation slope (ESPVR); (**f**) the maximal rate of relaxation (−d*P*/d*t*); (**g**) relaxation time constant calculated by Weiss method (τ); (**h**) end-diastolic pressure–volume relation (EDPVR). Data are presented as means ± SE. *compared between WT and TG mice; ǂ compared between mice with or without treatment. * or ^ǂ^ *P* <0.05; ** or ^ǂǂ^ *P*<0.01

Mouse left ventricular function was assessed using catheter based P-V loop measurements. The measurements exhibit the P-V loops of each mouse ventricle showing the volume-dependent pressure changes (Fig. 2a and b). These data indicate that the blood filling in the diastole stage is limited in RCM mice and the situation is improved in the RCM mice after the treatment with EGCg (Fig. 2b). Furthermore, we analyzed the pressure volume relationship (PVR) at end systole (upper line) and end diastole (Lower line) in Fig. 2a since the PVR at end systole or end diastole reflects the muscle internal contraction or relaxation status without the influence of the volume changes. Our data indicate that no significant changes are observed in end systole PVR (ESPVR) between WT and RCM mice before and after the treatment of EGCg. However, a significant change, increase, has been observed in RCM mice compared to the WT mice (Fig. 2a). After the treatment of EGCg, the increased end diastole PVR (EDPVR) is significantly reduced (Fig. 2a). These data indicate that increased left ventricular pressure in RCM is caused by the increased ventricular muscle internal pressure and green tea extract EGCg is able to reverse the increased ventricular internal pressure and improve diastolic function in RCM mice. Further analyses of systolic parameters such as ejection fraction (EF)(Fig. 2c), dp/d*t* max (Fig. 2d) and ESPVR (Fig. 2e) indicate that no significant changes have been observed in systolic function between WT and RCM mice before and after the treatment of EGCg. However, diastolic parameters such as –dp/dt min (Fig. 2f), Tau (Fig. 2g) and EDPVR (Fig. 2h) are significantly altered in RCM mice compared to WT mice and EGCg can correct the changed diastolic parameters in RCM mice.

### β-adrenergic stimulation cannot change cardiac filling and diastolic function in RCM

**Fig. 3:**
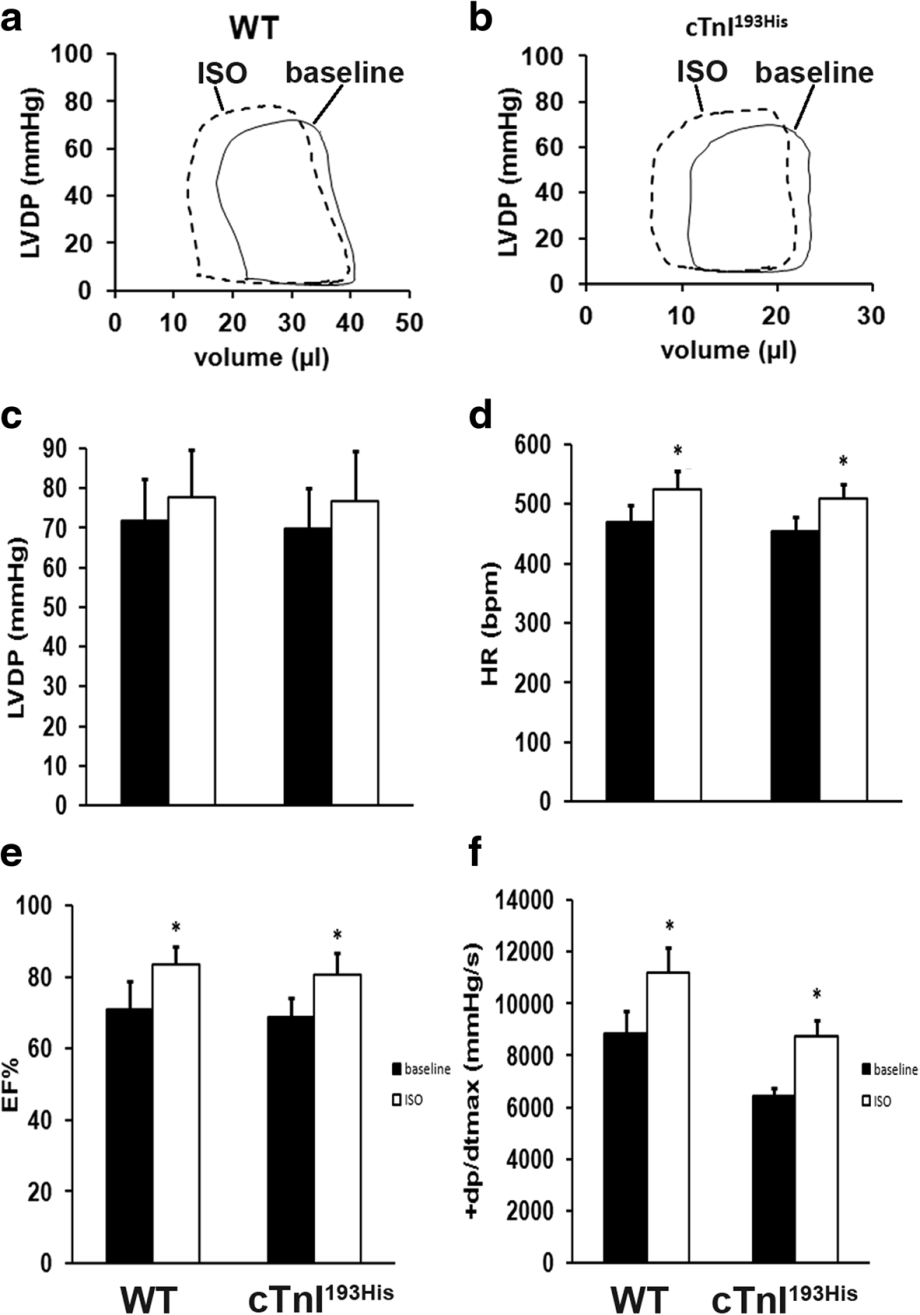
Inotropic responses to β-adrenergic stimulation in WT and cTnI^193His^ RCM mice. Representative pressure-volume loops recorded at baseline (solid curve) and after administration of isoproterenol (ISO; dashed curve) in WT mice (**a**) and in cTnI^193His^ RCM mice (**b**). **c** Left ventricular developed pressure (LVDP); (**d**) the heart rate (HR); (**e**) Ejection fraction (EF); (**f**) the maximal rate of contraction (+d*P*/d*t max*). Data are presented as means ± SE. *Significant difference compared between before and after ISO stimulation. * *P* <0.05

We have further tried to determine the effect of β-adrenergic stimulation in RCM mice by injection of isoproterenol (ISO) in tested mice. The data indicate that ISO treatment cannot increase the heart filling and has no effect on LVDP in RCM mice (Fig. 3a, b and c). However, ISO can stimulate heart rate (HR)(Fig. 3d), increase ejection fraction (EF)(Fig. 3e) and maximum contractility (Fig. 3f) in both WT and RCM mice, suggesting that ISO cannot change the internal muscle rigidity and cannot improve diastolic function in RCM mice.

### EGCg treatment does not alter Ca^2+^ handling proteins and their phosphorylation status

**Fig. 4:**
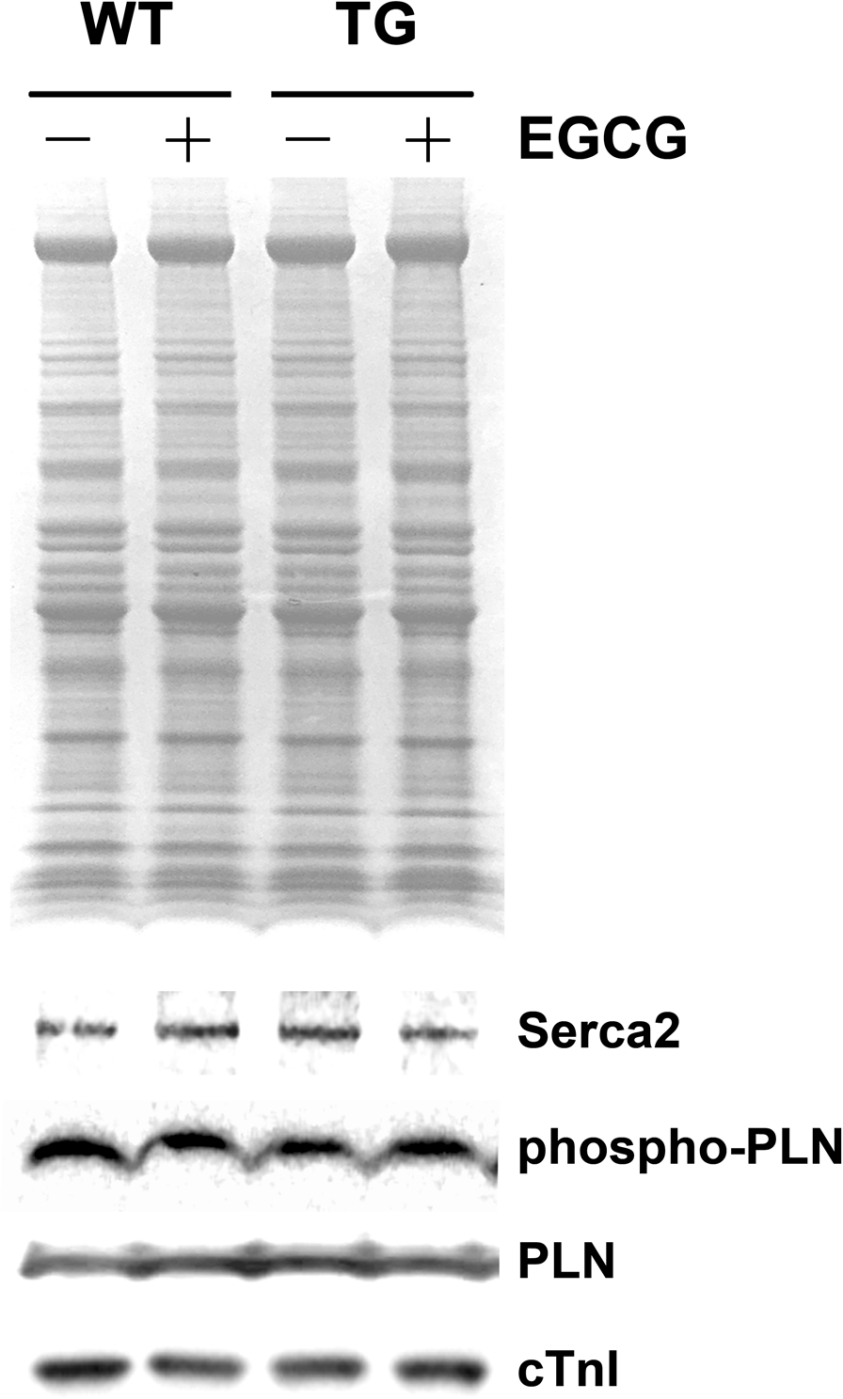
Levels of Ca^2+^ handling proteins and their phosphorylation status in myocardial cells with or without treatment of EGCg. A representative Western blot showing the levels of Ca^2+^ handling proteins and their phosphorylation status in WT and TG myocardial cells with or without EGCg treatment

Western blotting data indicate that the levels of the tested Ca^2+^ handling proteins do not show any significant changes both in WT and RCM mice after the treatment of green tea extract EGCg (Fig. 4). These proteins include the sarcoendoplasmic reticulum calcium transport ATPase (SERCA2), phospholamban (PLN) and phosphorylated phospholamban (phosphor-PLN). These data suggest that green tea extract EGCg corrects diastolic dysfunction and improve cardiac relaxation in RCM not via the alteration in Ca^2+^ handling protein and Ca^2+^ concentration in myocardial cells.

## Discussions

Heart failure (HF) is the leading cause of mortality in the United States [1]. In two major types of HF, HF with reduced ejection fraction (HFrEF) and HF with preserved ejection fraction (HFpEF), prevalence of HFpEF is rising, with mortality, morbidity, and healthcare costs now equal to those for HFrEF [2, 5, 15]. However, the pathophysiology of HFpEF is poorly understood, and no medication trials have had positive effects on their primary end-points. A major cause of HFpEF is diastolic dysfunction which is commonly seen in primary hypertension and various cardiomyopathies such as HCM and RCM. Diastolic dysfunction occurs when the ventricles cannot fill normally, and in severe conditions, it can lead to diastolic heart failure (DHF). Recently studies have revealed that cardiac diastolic dysfunction increases with advancing age, approximately 30–40 % of the population (aged 45 or older) had diastolic dysfunction, and nearly half of them were healthy individuals [16–18].

In the present study, we have further confirmed that diastolic dysfunction occurs in RCM mice with troponin mutations in the heart and desensitizing green tea extract EGCg can reverse the diastolic dysfunction in RCM hearts. Using micro-catheter based P-V loop measurements in both WT and RCM TG mice, we have successfully analyzed P-V loops and end diastolic pressure and volume relationship (EDPVR) in the experimental mice. The P/V index reflects the elastance of the left ventricles. The P/V plus dP/d*t* are common physiologic measurement of cardiac tissues. EDPVR is an index that reveals the internal ventricular muscle dynamics and developed pressure in diastole. Our data indicate that the EDPVR increased significantly in RCM heart compared to the WT hearts, suggesting an increased ventricular muscle internal pressure (force) that is Ca^2+^ independent in RCM hearts with troponin mutations. The increased ventricular muscle internal pressure (force) causes a rigid ventricle and enlarged left atrium or both atria, which are consistent with what we reported previously of cellular and morphological examination on the same RCM mice [10, 11]. Green tea desensitizing EGCg is able to reverse the diastolic dysfunction in RCM mice because it can correct the increased EDPVR in these mice without any significant effects on Ca^2+^ dynamics in myocardial cells by altering Ca^2+^ handling proteins or their phosphorylation status.

This is the first study investigating the ventricular muscle internal dynamics and pressure developed during diastole in RCM hearts with troponin mutations. It is also novel to demonstrate that green tea desensitizing EGCg reverse diastolic dysfunction by correcting the internal EDPVR in the RCM hearts. It is interesting to note that a beta-adrenergic agonist has barely any effect on diastolic function and heart filling although it can increase heart rate and ejection fraction in RCM mice.

The results from this study and our previous studies indicate that myofibril hypersensitivity is a key factor that causes impaired relaxation in HCM or RCM hearts associated with myofibril protein troponin mutations in myocardial cells. Green tea extract EGCg can reverse the impaired relaxation by correcting the hypersensitivity-induced abnormal ventricular muscle internal pressure in RCM hearts. Recently, the desensitization effect of green tea extracts has been further confirmed in cells from a patient with HCM [19]. The effect of green tea extracts occurs in internal ventricular muscle dynamics by interfering with calcium binding to troponin C in myocardial cells [20], and probably not by altering calcium dynamics through changes in calcium handling protein concentrations or their activities. However, one in vitro study using isolated murine cardiomyocytes reported that nanomolar EGCG could increase calcium transients [21]. Further studies are needed to clarify the effects of EGCG on calcium dynamics. In addition, it should be pointed out that diastolic dysfunction covers a vast range of etiology and causes, and green rea desensitization may not be so effective when it is applied to some types of diastolic dysfunction such as those related to fibrosis or amyloidosis.

## Conclusion

Green tea extract catechin is effective in correcting diastolic dysfunction and improving ventricular muscle intrinsic compliance in RCM caused by troponin mutations.

## Abbreviations

A, mitral Doppler A peak velocity; E, mitral Doppler E peak velocity; EDD, end diastolic dimension; EF, ejection fraction of LV; ESD, end systolic dimension; FS, fractional shortening of LV; HCM, hypertrophic cardiomyopathy; IVCT, isovolumetric contraction time; IVRT, isovolumetric relaxation time; IVS, intra-ventricular septum; LA, left atrium; LV, left ventricle; LVID:d, left ventricular internal diameter end diastole; LVID:s, left ventricular internal diameter end systole; PW, posterior wall thickness of LV; RA, right atrium; RCM, restrictive cardiomyopathy
